# Biological effects of combined resveratrol and vitamin D3 on ovarian tissue

**DOI:** 10.1186/s13048-017-0357-9

**Published:** 2017-09-15

**Authors:** Francesca Uberti, Vera Morsanuto, Silvio Aprile, Sabrina Ghirlanda, Ian Stoppa, Andrea Cochis, Giorgio Grosa, Lia Rimondini, Claudio Molinari

**Affiliations:** 1 Physiology Laboratory, Department of Translational Medicine, UPO, Via Solaroli, 17 28100 Novara, Italy; 2 Department of Pharmaceutical Sciences and Drug and Food Biotechnology Center, UPO, Novara, Italy; 3 Department of Health Sciences, Medical School, UPO, Novara, Italy

**Keywords:** Resveratrol, Vitamin D3, Phytoalexins, Hot flashes, Bioavailability

## Abstract

**Background:**

Resveratrol (3,5,4′-trihydroxy-trans-stilbene) is a natural antioxidant polyphenol able to exert a wide range of biological effect on several tissues. Despite its important beneficial properties, it has a low water solubility, which limits its therapeutic applications in humans. Resveratrol also acts as a phytoestrogen that modulates estrogen receptor (ER)-mediated transcription. In addition, it has been shown that ovarian tissues benefit greatly from vitamin D3, which exerts its beneficial effects through VDR receptors.

The aim was to evaluate the cooperative effects of resveratrol combined with vitamin D3 on ovarian cells and tissues and some other organs as well. Moreover, the modulation of specific intracellular pathways involving ER and VDR receptors has been studied.

**Methods:**

The experiments were performed both in vitro and in vivo, to analyze cell viability, radical oxygen species production, signal transductions through Western Blot, and resveratrol quantification by HPLC.

**Results:**

Cell viability, radical oxygen species production, and intracellular pathways have been studied on CHO-K1 cells. Also, the relative mechanism activated following oral intake in female Wistar rats as animal model was investigated, evaluating bioavailability, biodistribution and signal transduction in heart, kidney, liver and ovarian tissues. Both in in vitro and in vivo experiments, resveratrol exerts more evident effects when administered in combination with vitD in ovarian cells, showing a common biphasic cooperative effect: The role of vitamin D3 in maintaining and supporting the biological activity of resveratrol has been clearly observed. Moreover, resveratrol plus vitamin D3 blood concentrations showed a biphasic absorption rate.

**Conclusions:**

Such results could be used as a fundamental data for the development of new therapies for gynecological conditions, such as hot-flashes.

**Electronic supplementary material:**

The online version of this article (10.1186/s13048-017-0357-9) contains supplementary material, which is available to authorized users.

## Background

Resveratrol (3,5,4′-trihydroxy-trans-stilbene, RES) is a natural antioxidant polyphenolic compound belonging to stilbene phytoalexins, a sub-group of non-flavonoid phenolic compounds [1, 2]. RES is contained in various vegetables such as berries, grapes, peanuts, besides red wine [3]. Particularly, red wine is the main source of RES, but a recent study discovered that peanut sprouts contain abundant RES both in *cis* and *trans* isoforms [4, 5]. Cis- and trans-isomers of RES coexist in plants and in wine. RES is rapidly metabolized in vivo and has a low water solubility, which reduces the rate-absorption in cells [6] reducing oral bioavailability [7]. The effectiveness of orally administered RES depends on its absorption, metabolism and tissue distribution. At intestinal level, RES is absorbed by passive diffusion or through the formation of complexes with membrane transporters, whereas in the bloodstream it can be found as glucuronide, sulfate, or free as well [8]. In some studies performed on animal models, the peak concentrations of trans-RES occur in blood and serum very rapidly, about 15 min from the beginning of the administration [9]. Human studies on the absorption and bioavailability of RES used a single oral dose of 25 mg [10, 11], but it was difficult to detect non-metabolized RES in circulating plasma. In some studies, RES solubility has been increased by the use of ethanol (50 mg/mL) or other organic solvents [9]. Moreover, researchers have recently attempted to improve RES chemical stability, water-dispersibility, bioavailability, permeability through blood–brain barrier (BBB) and therapeutic efficacy by using nanostructure-based drug delivery systems [12–14]. A number of epidemiologic studies have shown that RES has beneficial effects in preventing various pathologic conditions ranging from cardiovascular diseases to cancer [15]. As reported by in vitro studies, RES can inhibit cell proliferation, induce apoptosis and block cell cycle progression in numerous types of human cancer cell lines, such as those of the colon, skin, breast, lung, prostate and liver, as well as pancreas [10]. In addition, in a few in vivo experimental models of colon and esophagus cancers the effectiveness of oral doses of RES was shown [16, 17]. RES acts as a phytoestrogen modulating estrogen receptor (ER)-mediated transcription [18]. The estrogenic role of RES is important since a variety of RES-sensitive tissues are ER-positive and the two ER subtypes in mammals, ERα and ERβ, exhibit different tissue-specific expression profiles [19]. Specifically, effects of RES on ER include anti-inflammatory effects such as protection from trauma-hemorrhage-induced injury and suppression of Interleukin-6 (IL-6) expression in the liver, intestine and cardiovascular system [20]. However, in contrast to other ERα agonists, resveratrol does not induce proliferation of mammary or uterine tissues, allowing it to be taken as a dietary supplement. RES binds and increases the transcriptional activity of estrogen receptors (ERα and ERβ) at 50–100 μM [19–22]. RES displays a great affinity for ER behaving as either agonist or antagonist in a cell- and tissue-specific manner [23]. This is important to explain the effectiveness of RES in reducing the number of vasomotor episodes and the intensity of hot flashes (HF), with the transition from moderate/severe to mild symptoms in 78.6% of patients [24]. RES has the characteristics to be an alternative therapy in the treatments of HF in menopause. The common incidence of hot flashes is around 75% and presently hormone replacement therapy is the gold standard in the management of moderate to severe vasomotor symptoms associated with menopause. RES has also been associated with anti-inflammatory effects, particularly in tissues that contain a large number of estrogen receptors, through this connection has been studied, but there are few studies on the mechanisms activated [25, 26].

In recent years, vitamin D has seen growing interest among researchers, especially due to the presence of its receptor (VDR) in many tissues and organs. It has been demonstrated that in ovarian tissues a high density of VDR is present as well [27, 28] and vitamin D3 (the active form of vitamin D, 1,25-dihydroxyvitamin D3, vitD) acts through intracellular mechanisms similar to what observed for RES [29]. The role of vitD in cellular growth regulation is demonstrated by its ability to arrest cells in the G1/G0 phase of the cell cycle, and by up-regulating p21, a powerful tumor suppressor gene. Thus, vitD can control cell division and proliferation [30]. VitD also has important anti-proliferative, anti-angiogenic and pro-differentiative effects in a wide range of cancers [31]. Interestingly enough, many of the bioeffects of resveratrol overlap with reported benefits from high circulating levels of vitD. Thus, given the ability of vitD to elicit a wide range of bio-effects via transcriptional regulation, evaluating resveratrol in the context of VDR signaling is of particular interest to help in elucidating the molecular pathways involved by these two dietary lipophilic substances in optimizing healthspan and well aging [30]. The potential for resveratrol to modulate vitamin D receptor signaling has recently been postulated [32, 33]. There is an overall structural symmetry and parallel configuration of resveratrol and known VDR ligands, which could suggest that resveratrol might serve as a low-affinity VDR ligand with the ability to activate VDR. Intriguingly, several targets emerge such as eNOS, cyclooxygenase, and Akt kinase, all of which are likewise regulated by vitD [34]. VitD can exert its beneficial effects through several important signaling pathways mediated through genomic and non-genomic mechanisms [35]. Finally, vitD exerts beneficial effects on ovarian tissues preventing ROS-derived cellular injury [28]. Therefore, since these two substances have similar effects on ovarian cells, some form of interaction in exerting effects can be hypothesized. This could lead to interesting results for future clinical use in menopause-related conditions like hot flashes. For this reason, the aim of this study was to evaluate the biological effects of RES combined with the active form of vitD in order to increase the absorption of RES using vitD that it is able to activate the same intracellular pathways of RES on cultured ovarian cells and tissue.

## Methods

### Cell culture

CHO-K1 (Chinese Hamster Ovary cell), purchased from Lonza (Basel), were cultured in Dulbecco’s modified Eagle’s medium: Nutrient Mixture F-12 (DMEM-F12; Sigma, Milan, Italy) supplemented with 10% fetal bovine serum (FBS, Sigma, Milan, Italy), 2 mM glutamine and 1% penicillin/streptomycin (Sigma, Milan, Italy) and incubated at 37 °C, 5% CO_2_, and 95% humidity [36]. When the cells reached 80–90% of confluence were seeded for different experiments; 1 × 10^4^ and 2.5 × 10^4^ cells were plated in a 96-well for MTT test and ELISA activation assay, respectively; 1 × 10^5^ cells plated on 24-well plates to analyzed ROS production; for Western blot analysis and SOD activity the cells were seeded in 6 wells and maintained until 85% of confluence.

### Experimental protocol in vitro

The cells before treatments were placed overnight in Dulbecco’s modified Eagle’s medium (DMEM; Sigma, Milan, Italy) without red phenol and FBS in incubator at 37 °C, 5% CO_2_, and 95% humidity. The cells were treated with a range of Resveratrol (RES) 10-100 μM to determine an optimal concentration; 50 μM was chosen and its efficacy verified in a time-course study (from 2 min to 48 h). The RES concentration was chosen basing on previous studies about the therapeutic range of ovarian evidence [37, 38] and on the experiments of dose-response study. RES was prepared in lipidic solvent that was also tested alone in CHO-K1 cultures. The cooperative activity of RES with vitD (active form of vitamin D, 1,25-dihydroxyvitamin D3), was also tested, evaluating the effects of the co-stimulation with RES 50 μM and vitD 100 nM [39] in CHO-K1 cells during time.

### MTT test

MTT-based In Vitro Toxicology Assay Kit (Sigma-Aldrich) was performed as described in literature [40] to determine cell viability after stimulations. Cells were incubated in DMEM without red phenol 0% FBS with 1% MTT dye for 2 h at 37 °C in incubator [41] and then cell viability was determined measuring the absorbance through a spectrometer (VICTORX4 multilabel plate reader) at 570 nm with correction at 690 nm. The results were obtained comparing the results to control cells (100% viable).

### ROS production

The rate of superoxide anion production was determined as a superoxide dismutase-inhibitable reduction of cytochrome C, following a standard technique [41, 42]. In both treated and untreated cells, 100 μl of cytochrome C were added and in another sample, 100 μL of superoxide dismutase were also added for 30 min in incubator (all substances from Sigma-Aldrich). The absorbance was measured at 550 nm by spectrometer (VICTORX3 Multilabel Plate Reader) and the O_2_ was expressed as nanomoles per reduced cytochrome C per microgram of protein.

### Akt/ERK activation assay

The InstantOne™ ELISA is specifically engineered for accurate measurement of phosphorylated ERK 1/2 and AKT in cell lysates, following the manifacturer’s instructions (Thermo-Scientific). Cells at the end of treatments were lysated with 100 μL Cell Lysis Buffer Mix, shaken for 10 min at RT and 50 μL/well of each sample were tested in InstantOne ELISA microplate strips including the 50 μL/well Positive Control Cell Lysate and 50 μL/well negative control. At each well 50 μL of prepared Antibody Cocktail were added and the strips incubated for 1 h at room temperature on a microplate shaker and washed 3 times with 200 μL/well of Wash Buffer (1X). At the end, 100 μL of the Detection Reagent were added to each well and after 20 min the reaction was stopped adding to each well 100 μL of Stop Solution. The strips were measured by a spectrometer (VICTOR X4 multilabel plate reader) at 450 nm. The results were expressed as means Absorbance (%) compared to control.

### SOD activity assay

Cayman’s Superoxide Dismutase Assay Kit utilizes a tetrazolium salt for detection of superoxide radicals generated by xanthine oxidase and hypoxanthine. The SOD assay measures all three types of SOD (Cu/Zn, Mn, and FeSOD). The cells and tissue were lysed after treatments following manufacturer’s instructions (Cayman). In a 96 well, at every sample of 10 μl were added 200 μl of the diluted Radical Detector. At the same time, a standard curve was prepared (0.05–0.005 U/ml). Then, 20 μl of diluted Xanthine Oxidase were added at all wells and the plate mixed for 30 min at RT and then the absorbance measured through a spectrometer (VICTOR X4 multilabel plate reader) at 480 nm. The results were expressed as a means (%) compared to control.

### Western blot of cell lysates

CHO-K1 cells were lysed in ice Complete Tablet Buffer (Roche) supplemented with 2 mM sodium orthovanadate, 1 mM phenylmethanesulfonyl fluoride (PMSF; Sigma-Aldrich), 1:50 mix Phosphatase Inhibitor Cocktail (Sigma-Aldrich) and 1:200 mix Protease Inhibitor Cocktail (Calbiochem). 35 μg of proteins of each sample were resolved on 10% SDS-PAGE gel. Polyvinylidene difluoride membranes (PVDF, GE, Healthcare Europe GmbH, Milan, Italy) were incubated overnight at 4 °C with specific primary antibody: anti-VDR receptor (1:400, Santa-Cruz) and anti-ERβ (1:500, Santa-Cruz). Protein expression was normalized to the specific total protein (if possible) and verified through β-actin detection (1:5000; Sigma-Aldrich) and expressed as a mean ± SD (%).

### Animal model

Female Wistar rats weighing 300 to 350 g (*n* = 94) purchased from Envigo^++++^ (Bresso, Italy), were housed in a room at a constant temperature of 25 °C on a 12-h/12-h light/dark cycle with food and water available ad libitum. All experiments were conducted in accordance with local ethical standards and prospectively approved by the University OPBA (*Organismo Preposto al Benessere degli Animali*, Animal Wellness Committee). Experimental protocols were approved by national guidelines (Ministero della Salute authorization number 914/2015-PR) and in accordance with Guide for the Care and Use of Laboratory Animals (National Institutes of Health publication 86–23, 1985 revision).

### In vivo experimental protocol

In order to study the bioavailability 0.5 mg RES were administrated by gavage following a standard technique [43, 44]; the quantity of RES was calculated by the conversion formula (animal-man) approved by FDA [45]. For each animal, anesthesia was performed via isoflurane (1.2–1.5 Mean Alveolar Concentration) in oxygen and gavage was carried out using probe-ended stainless-steel gastric tubes (80 × 1.5 mm, length × outer diameter). After treatment, rats were placed in individual cages and housed separately for the duration of the study and daily monitored. The animals were randomized in different groups: *n* = 36 treated with RES lipophilic formula; n = 36 with RES plus vitD 0.4 μg lipophilic formula; *n* = 18 treated with vitD 0.4 μg alone; *n* = 4 untreated (control) and sacrificed at T0. Time-point for each treatment (2, 5, 15, 30, 60, 180, 360, 720, 1440 min) was conducted in triplicates. The animals were euthanized by CO_2_ asphyxiation at each time point and the organs (liver, stomach, intestine, heart, kidneys and ovaries) were withdrawn to evaluate biodistribution of the different RES formulations, and to evaluate the ovarian tissue integrity by Western-blot. In addition, blood samples used for RES determination by HPLC analysis, the oxygen radical species (ROS) and vitD quantification (by ELISA kit) were collected at each time-point using CBC tubes to obtain plasma by centrifugation at 3000 rpm for 15 min at room temperature.

### Plasma vitamin D quantification

Vitamin D3, the active form of vitamin D, is very short-lived and rapidly metabolized to the deactivated forms 24,25(OH)2D3 and 1,24,25(OH)3D3. For this reason a competitive EIA assay kit has been used that primarily detects the more metabolically stable forms, 25(OH)D3 and 25(OH)D2 (Cayman’s Vitamin D EIA Kit). At the end of each time point, plasma samples were collected using EDTA-Na2 as an anticoagulant, centrifuged for 15 min at 1000×g at 4 °C within 30 min and then the supernatant used immediately. Before adding to wells, the SABC working solution and TMB substrate were equilibrate for at least 30 min at room temperature and the strips of the plate washed twice before adding standard, sample and control. For the quantification it is necessary to plot a standard curve including control (zero well). 0.1 ml of each sample and standard were added into test sample wells, the plate sealed with a cover and incubated at 37 °C for 90 min. After the plate content was removed, 0.1 ml of Biotin- detection antibody work solution was added into the standard and test sample at 37 °C for 60 min. After the plate was washed 3 times with Wash buffer 0.1 ml of SABC working solution into each well was added and the plate incubated at 37 °C for 30 min. After the plate was washed 3 more times with Wash buffer 90 μl of TMB substrate into each well was added and the plate incubated at 37 °C in dark within 15–30 min. After this time 50 μl of Stop solution into each well was added and the absorbance measured at 450 nm in a microplate reader immediately after adding the stop solution.

### Total plasma antioxidant capacity

The concentration of radical oxygen species (ROS) in plasma was measured in a 96-well plates using the Antioxidant Assay kit (Cayman) following the manufacturer’s instructions [46]. In brief, 10 μl of Metmyoglobin and 150 μl of Chromogen per well were added in plasma and standard samples (Trolox in Assay buffer from 0 mM to 0.33 mM) and the reactions started adding 40 μl of Hydrogen Peroxide Working Solution to all the wells. The 96-well plate was covered, mixed for 5 min at room temperature and the absorbance was measured using spectrophotometer (VICTORX4 Multilabel Plate Reader) at 750 nm or 405 nm. The results were expressed as means ± SD (%).

### Western-blot of ovarian tissues

Ovarian tissues were immediately washed with ice 0.9% saline solution (*w*/*v*), weighed and homogenized in a volume of 100 mg tissue/300 μL of lysis buffer (0.1 M Tris, 0.01 M NaCl, 0.025 M EDTA, 1% NP40, 1% Triton X100, Sigma-Aldrich, Milan) supplemented with 2 mM sodium orthovanadate, 0.1 M sodium fluoride (Sigma-Aldrich, Milan), 1:100 mix of protease inhibitors (Sigma-Aldrich, Milan), 1:1000 phenylmethylsulfonyl fluoride (PMSF; Sigma-Aldrich, Milan), using an electric potter at 1600 rpm for 2 min. Samples were mixed for 30 min at 4 °C, centrifuged for 30 min at 13000 rpm at 4 °C and 40 μg of proteins for each samples resolved on SDS-PAGE gel at 15%. Proteins transferred to polyvinylidene fluoride membranes (PVDF, GE Healthcare Europe GmbH, Milan, Italy) were incubated overnight at 4 °C with specific primary antibody: anti-VDR receptor (1:400, Santa-Cruz), anti-ERβ (1:500, Santa-Cruz), anti-cyclin-D1 (1:1000, Euroclone, Milan, Italy). Protein expression was normalized and verified through β-actin detection (1:5000; Sigma-Aldrich) and expressed as a mean ± SD (%).

### RES quantification in CHO-K1

At the end of stimulations cells were placed in ice and supernatants were collected in 1.5 ml centrifuge tubes to determine the rate of extracellular RES. 1 × 10^6^ CHO-K1 cells at the end of stimulations were washed with cold 0.9% saline solution, lysed in ice 0.9% saline solution, mixed for 10 min at 4 °C and centrifuged for 20 min at 13000 rpm at 4 °C. Supernatants were used for quantification of intracellular RES. Samples were diluted with equal volume of acetonitrile, vortexed, centrifuged at 13000 rpm for 10 min, and analyzed by HPLC-UV (Additional file 1).

### RES quantification in rat plasma and tissues

RES quantification in rat plasma and tissue samples (liver, stomach, intestine, heart, kidneys and ovaries) was carried out by HPLC-MS analysis. Tissues were homogenized in a volume of 100 mg tissue/300 μl of ice 0.9% saline solution (*w*/*v*) at 1600 rpm for 2 min and the lysates were mixed for 20 min at 4 °C, and then centrifuged at 13000 rpm for 30 min at 4 °C. Plasma and tissue supernatants were processed as follows. An aliquot of 50 μl of plasma or tissue sample was mixed with 50 μl of 1 M of sodium acetate buffer (pH = 5.5) and 2.5 μl of β-glucuronidase/arylsulfatase from *Helix pomatia* in a 1.5 ml centrifuge tube*.* Ethyl acetate (600 μl) was added, then sample was extracted by vortexing (40s), and centrifuged at 13000 rpm for 10 min. An aliquot (550 μl) of the organic layer was transferred into 1.5 ml centrifuge tube and evaporated at 45 °C under reduced pressure for 40 min. The residue was dissolved in 100 μl of acetone containing the IS (trans-4-hydroxystilbene- final concentration, 200 μg/l), 25 μl of 0.1 N NaOH, and 100 μl of 1 mg/ml Dns-Cl (dansyl chloride) solution in acetone. Sample was shortly vortexed and heated at 45 °C for 20 min. After centrifugation (13,000 rpm for 5 min) the sample was analysed by HPLC-MS (Additional file 1). Calibration curve for RES quantification was prepared by spiking blank matrixes and processed as described above, except for the addition of β-glucuronidase/arylsulfatase.

### Statistical analysis

In vitro results obtained from at least 5 independent experiments conducted in triplicates were expressed as means ± SD, using One-way ANOVA followed by Bonferroni post hoc test. Values of significance for *p* < 0,05 were considered statistically significant. Data collected from in vivo results obtained from 4 independent experiments were analyzed by two-way ANOVA and one-way ANOVA followed by Bonferroni post hoc test and the comparisons between the two groups were performed using a two-tailed Student’s t-test. Multiple comparisons between groups were analyzed by two-way ANOVA followed by a two-sided Dunnett post-hoc testing. *P*-value < 0.05 was considered statistically significant.

## Results

### Dose-response and time-course study

**Fig. 1:**
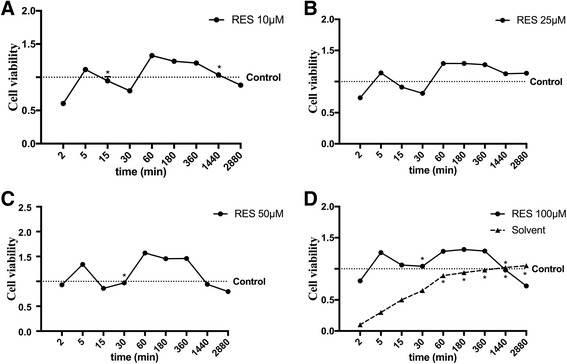
Time-course and dose-response study of CHO-K1 viability measured by MTT test. **a** time-course of RES 10 μM; **b** time-course of RES 25 μM; **c** time-course of RES 50 μM; **d** time-course of RES 100 μM. Reported data are means ± SD of five independent experiments. * not significant vs control; point without symbol *p* < 0.05 vs control. RES = resveratrol. The effect of solvent alone is reported as well

A dose response and a time-course study were planned to identify the dose of Resveratrol (RES) able to induce the maximal effect on cell viability during time. In addition, these experiments were important to understand the cooperative effect of RES with vitamin D3 (vitD) during time. As shown in Fig. 1c RES 50 μM appeared to be the dose producing the greatest effect (*p* < 0.05) compared to control and to other concentrations (10, 25, 100 μM) during all time periods considered (Fig. 1a–d). This concentration of RES was maintained for all successive experiments. In addition, another important finding regarded the reaction time of RES 50 μM, which appeared as a biphasic curve that quickly started (at 2 min), confirming its rapid metabolism. Its beneficial effect was maintained as long as 3 h.

**Fig. 2:**
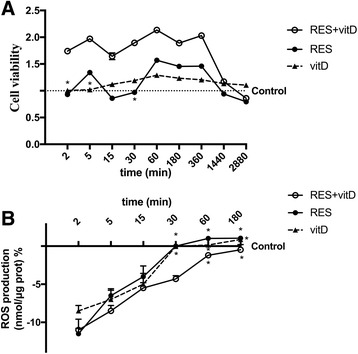
The cooperative effect of RES and vitD during time on cell viability and ROS production in CHO-K1 cells. **a** cell viability and **b** ROS production measured during time-course study in presence of RES and vitD alone and combined. Reported data are means ± SD in (**a**) and they are expressed as means ± SD(%) in (**b**) of five independent experiments. * not significant vs control; points without symbol, *p* < 0.05 vs control. RES = resveratrol 50 μM; vitD = vitamin D3 100 nM; RES + vitD = co-stimulation of RES 50 μM with vitD 100 nM

The combination of RES 50 μM with vitD 100 nM was able to amplify biological effects with a similar kinetic reaction to RES alone (*p* < 0.05); vitD was already able to stabilize and enhance the effect of RES from 2 min of treatment (Fig. 2a). Then we observed a stable plateau phase around 6 h and then effects began to decline for the following 48 h. For this reason, we have chosen to study the kinetics ranging from 2 min to 3 h for all successive experiments. As shown in Fig. 2b, the radical oxygen species (ROS) produced by RES alone and combined with vitD appeared to be modulated during time. In particular, in the first minutes (from 2 to 15 min) after treatment with RES alone a significant ROS production compared to control (*p* < 0.05) was observed and the presence of vitD was able to amplify this effect. This combination was also able to maintain a reduction of ROS up to 1 h of stimulation (*p* < 0.05); at 3 h ROS production was comparable to control (*p* > 0.05). These data confirm the beneficial effects previously observed on cell viability of CHO-K1 cells and the importance of the combination of RES and vitD to maintain the beneficial effects of RES during time.

### Quantification of intracellular RES with or without vitD3 in CHO cells

**Fig. 3:**
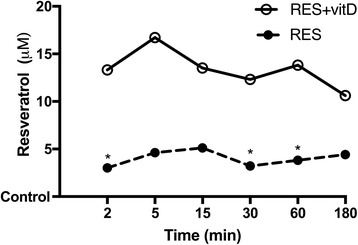
Measure of intracellular concentration of RES alone and combined with vitD in CHO-K1 cells in a time-course study. Reported data are means ± SD of five independent experiments. * not significant vs control; points without symbol, *p* < 0.05 vs control. RES = resveratrol 50 μM; RES + vitD = co-stimulation of RES 50 μM with vitamin D 100 nM

Since the biological effects of RES were due to its ability to be absorbed in cells and tissues, the intracellular concentration of RES in CHO-K1 during time was determined by HPLC-UV. As reported in Fig. 3, the absorption rate of RES combined with vitD was enhanced compared to RES alone (*p* < 0.05), in particular in the first 15 min of stimulation; the maximum effect was observed at 5 min of stimulation (about 16.7 μM). These findings confirmed previous data about the cooperative effect of RES plus vitD; vitD was important to amplify and stabilize the effects of RES influencing also the level of RES uptake in ovarian cells. Finally, this time range (from 2 to 15 min) of stimulation was used to verify the intracellular cascade activated by RES alone and combined with vitD.

### Analysis of the main intracellular pathways activated by RES and vitD

**Fig. 4:**
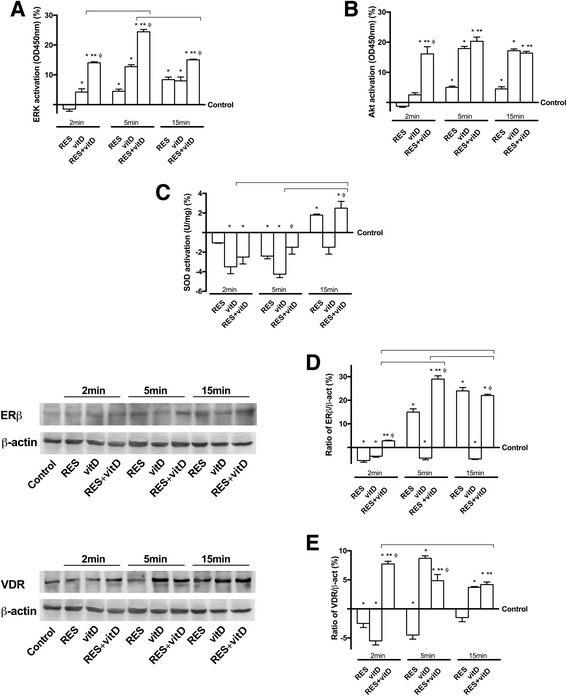
Western Blot, densitometric analysis and protein activation of CHO-K1 cells stimulated with RES and vitD alone and together. In **a** (ERK/MAPK), **b** (Akt), **c** (SOD) activations by ELISA are reported as means ± SD(%) of five independent experiments. In **d** (ERβ receptor) and **e** (VDR receptor) Western blot (on the left) and densitometric analysis (on the right) are reported. In the right column the specific densitometric analysis is reported and expressed as means ± SD(%) of five independent experiments. * *p* < 0.05 vs control; ** *p* < 0.05 vs RES alone; ^φ^ *p* < 0.05 vs vitD; the bars, *p* < 0.05 between RES + vitD at different times. RES = resveratrol 50 μM; vitD = vitamin D3 100 nM; RES + vitD = co-stimulation of RES 50 μM with vitD 100 nM

In order to assess which intracellular pathways were activated after intracellular uptake of RES alone and combined with vitD, ERKs, Akt, SOD, ERβ and VDR signaling were investigated in CHO-K1 cells. As reported in Fig. 4a and b, the role of MEK1/MAPK and PI3K/Akt pathways was examined to explain the action mechanism of RES alone and combined with vitD; MAPK and PI3K signal transduction pathways are closely associated with the one of healthy tissue. To study changes in the activation levels of proteins associated with cellular signal transduction according to treatment time, ELISA was performed following treatment for various time periods up to 15 min. The results confirmed an increase in activation of ERK and Akt due to RES alone and these effects were amplified by the presence of vitD. In particular, the results showed that the activation levels of ERK and Akt started at 2 min and the maximum effect was observed at 5 min in both RES alone and combined with vitD compared to control (*p* < 0.05) then decreased. These data confirmed the importance of vitD in amplifying the beneficial effects of RES to maintain healthy tissue. In addition, since the beneficial effects of RES included its anti-radical action, two important mechanisms involved such as SOD activity and ERβ, were also investigated by ELISA and Western blot respectively. As reported in Fig. 4c, SOD activity was maintained similar to control, demonstrating the ability of RES combined with vitD to maintain ROS production low during time. In addition, these anti-oxidant effects were obtained via ERβ activation (Fig. 4d); the involvement of ERβ was observed starting from 2 min after treatment with RES alone and increasing after 5 min compared to control (*p* < 0.05); the presence of vitD amplified (*p* < 0.05) all these levels of activations (about 20% at 2 min and 65% at 5 min of RES plus vitD compared to RES alone), indicating a cooperative activity exerted by RES and vitD. The importance of combined treatments with RES and vitD was also confirmed on VDR expression (Fig. 4e), in which a stable activation of VDR during time was observed (*p* < 0.05), indicating its involvement in the beneficial effects previously observed. Such results indicate that the maintenance of tissue health induced by RES is mediated through the ERK, Akt, SOD, ERβ and VDR signal transduction pathways, which can help clarify that these beneficial effects exerted by vitD are a necessary condition.

### Bioavailability of resveratrol with vitamin D3

**Fig. 5:**
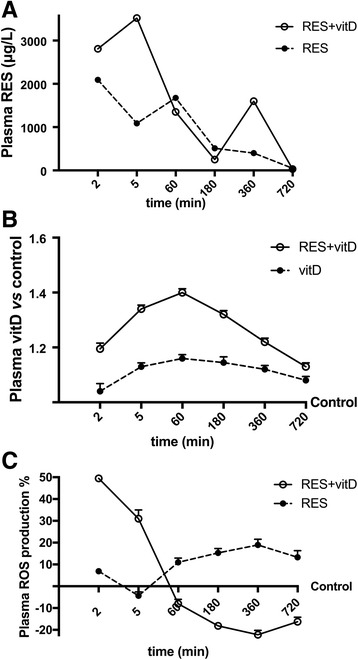
Bioavailability, vitamin D quantification and ROS production in in vivo experiments. Female rats (*n* = 48) were treated with RES 0.5 mg alone (*n* = 24), combined with vitD 0.4 μg (n = 24) and with vitD 0.4 μg alone (*n* = 18) by gavage. The animals were sacrificed at specific time-points (ranging 2–270 min) and plasma samples were collected. In **a** RES plasma concentration (μg/L), in **b** vitD plasma quantification and in **c** the ROS production are reported. The results are expressed as means ± SD in panel (**a**) and as means ± SD(%) in panel (**b**) of 4 independent experiments. All data *p* < 0.05 vs control. RES = resveratrol 0.5 mg; RES + vitD = preparation composed of RES 0.5 mg and vitD 0.4 μg

Since the biological effects of RES and vitD were reported in an in vitro study, some additional experiments were performed to demonstrate their efficacy in in vivo study as well, starting from bioavailability of RES alone and combined with vitD, following a time-course experiments (2, 5, 60, 180, 360, 720 min). As reported in Fig. 5a on rat plasma samples, the concentration of RES plus vitD and RES alone were time-dependent and followed by a biphasic curve. Plasma concentration started to enhance at 2 min (*p* < 0.05). The plasma of rats treated with RES alone showed a second peak of RES concentration at 1 h of treatment (about 20% lower than 2 min) and then the concentration decreased reaching control values. The presence of vitD significantly amplified the absorption rate of RES already in the first 2 min (about 34% compared to RES alone at 2 min) and obtained the maximum effects at 5 min (about 220% compared to RES alone at 5 min). In addition, a second peak was extended in time (after 1 h) because the absorption rate after 5 min was similar to what observed with RES alone as long as 3 h. At 6 h a second peak was shown and then the plasma concentration decreased leading to control values. This finding about the second peak supported the hypothesis that RES can be stored in organs to explain a secondary effect in the long run. All these data supported the importance of the cooperative activity of RES and vitD and explained the role of vitD in supporting the biological activity of RES. As reported in Fig. 5b, the quantification of vitD in plasma samples showed an increase in quantity of vitD present in plasma when vitD was administered with RES during time. These data confirmed the mutual influence of RES and vitD on the absorption after oral intake. In addition, the ROS concentration assessment in plasma of rats confirmed a positive influence of vitD on anti-radical mechanism induced by RES (Fig. 5b) during time; a slow and progressive decrease starting from 1 h of treatment with RES combined with vitD was observed and this effect is aligned with the absorption test at the time the maximum absorption rate has been reached. All these data explained the ability of RES plus vitD to rapidly cross the membrane and to reach target tissues.

### Analysis of the intracellular pathways activated in ovarian tissue

**Fig. 6:**
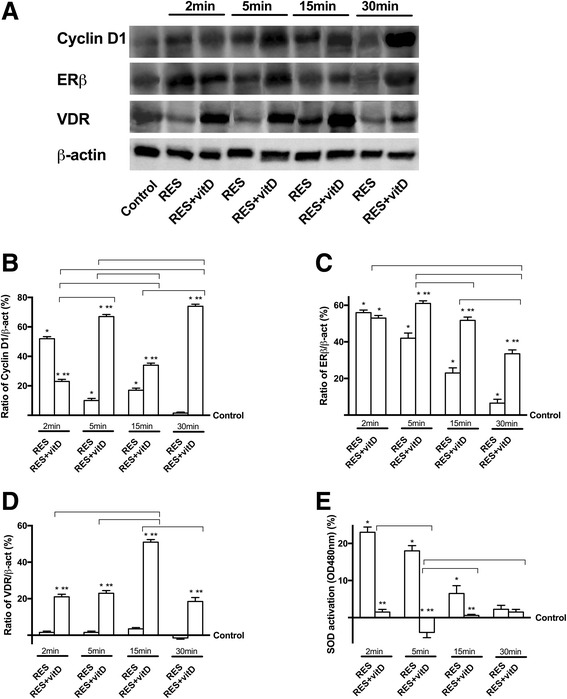
Western blot, densitometric analysis and protein activity of ovarian tissue obtained from female rats (*n* = 32) treated with RES alone (*n* = 16) and combined with vitD (n = 16). In the upper (**a**) an example of Western Blot taken at different time (ranging 2–30 min) of Cyclin D1, ERβ receptor and VDR receptor is reported. In the downstream the specific densitometric analysis of Cyclin D1 (**b**), ERβ receptor (**c**), and VDR receptor (**d**) is reported and expressed as means ± SD(%) of 4 independent experiments. In **e** SOD activity by ELISA was reported as means ± SD(%) of 4 independent experiments. * *p* < 0.05 vs control; ** *p* < 0.05 vs RES alone; the bars, *p* < 0.05 between RES + vitD at different times. RES = resveratrol 0.5 mg; RES + vitD = preparation composed of RES 0.5 mg and vitD 0.4 μg

In order to clarify the importance of bioavailability after oral intake of RES combined with vitD in gynecological disorders, some intracellular pathways involved in the biological effects of RES and vitD were also investigated in ovarian rat tissues during the first minutes (2, 5, 15, 30 min), following the plasmatic changes. As reported in Fig. 6, a better influence of RES plus vitD than RES alone (*p* < 0.05) has been demonstrated by the involvement of Cyclin D1 (Fig. 6a, b), an important regulator of G1 to S phase progression. The effects of RES plus vitD started at 5 min compared to RES alone (*p* < 0.05) and were maintained during time, indicating an improvement in cell cycle turn-over, important to maintain the integrity of tissue. This finding was supported by a decrease in SOD activity (Fig. 6e) observed with RES combined with vitD taken at the same time as Cyclin D1, compared to RES alone. These improvements of the biological effects of RES were obtained due to the presence of vitD, supporting previous data on the cooperative effects. The mechanism activated by RES plus vitD involved both ERβ (Fig. 6a, c) and VDR receptors (Fig. 6a, d), as reported. As a matter of fact, RES plus vitD showed the strongest effect on ERβ starting from 5 min (*p* < 0.05) compared to RES alone and on VDR starting from 2 min (*p* < 0.05) compared to RES alone. These effects were maintained during all time of stimulation. All these findings supported the in vitro results about the cooperative effect of RES and vitD on ovarian tissue.

### Biodistribution of resveratrol with vitamin D3

**Fig. 7:**
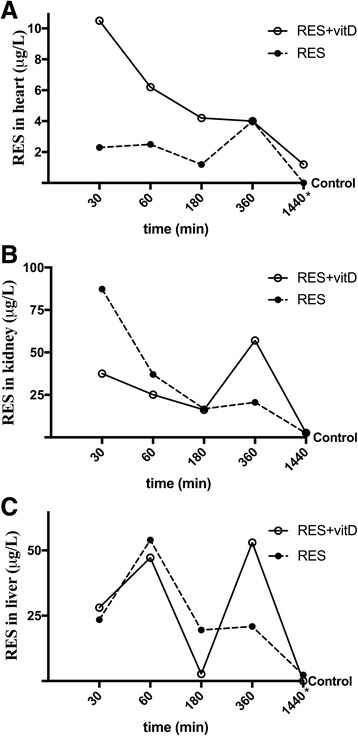
Biodistribution of RES (μg/L) in in vivo experiments. Female rats (*n* = 40) were treated with RES 0.5 mg alone (*n* = 20) and combined with vitD 0.4 μg (n = 20) by gavage. The animals were sacrificed at specific time-points (ranging 30–270 min) and heart (**a**), kidney (**b**) and liver (**c**) were collected. The results are expressed as means ± SD (μg/L) of 4 independent experiments. * not significant vs control; point without symbol, *p* < 0.05 vs control. RES = resveratrol 0.5 mg; RES + vitD = preparation composed of RES 0.5 mg and vitD 0.4 μg

Another important parameter useful to understand the biological effects of RES combined with vitD after oral intake and blood concentration was the biodistribution and accumulation of RES in different organs during time (30, 60, 180, 360, 720 min), such as heart (Fig. 7a), kidney (Fig. 7b), and liver (Fig. 7c). As reported, the absorption rate in tissue of RES alone and RES plus vitD was different and time-dependent, confirming the hypothesis about the activity of RES in the second peak observed in plasma samples. In addition, a better efficacy of RES combined to vitD (*p* < 0.05) in creating a tissue deposit of RES was confirmed mainly at 360 min in all organs tested (*p* < 0.05), indicating the importance of the combination with vitD to exert a systemic biological effect.

## Discussion

In the present study, it has been demonstrated that RES exerts more evident effects when administered in combination with vitD in ovarian cells, therefore showing a cooperative effect. Specifically, in CHO-K1 cells RES combined with vitD: a) showed biphasic biological effects; b) increased the number of viable cells; c) decreased ROS production; d) modulated, in a time-dependent manner, the levels of ERK/MAPK and Akt/PI3K. Resveratrol appeared to act involving ERK and Akt pathways via attenuation of ROS generation since it is combined with vitamin D3. These findings allow us to confirm the antioxidant effect of RES, which is mediated by SOD modulation, as shown by data collected in the in vitro experiments of this study. As regards the biphasic response observed, after the early effect, found at 2 min, a decrease was present probably due to the rapid metabolism of RES and then the effect on cell viability showed a significant rise that lasted as long as 3 h. To explain this finding, it can be hypothesized that an activation of long-latency intracellular metabolic pathways has occurred. As a matter of fact, a biphasic RES response has also been observed in other studies [47, 48]. The observation that beneficial effects of RES on cultured ovarian cells are enhanced by the co-stimulation with vitD is novel and important and it underlines the existence of a proper regulation essential to sustain tissue homeostasis.

Moreover, it is noteworthy that cooperative effects exerted by combined RES and vitD have been made possible through the concurrent involvement of ERβ and VDR receptors. This finding assumes great relevance for the ovarian tropism, since it has been demonstrated that in the ovary, RES exhibits antiproliferative and androgen-lowering effects on theca-interstitial cells [49]. RES exerts a cytostatic, but not cytotoxic effect in granulosa cells, while inhibiting aromatization and VEGF expression [49]. In addition, RES may increase the follicular reserve and extend the duration of ovarian life as an antiaging agent. The results of ongoing clinical trials are expected with impatience [50]. However, RES studies in ovarian physiology are limited. RES was reported to exert estrogenic effects, increasing the uterine and ovarian weight [51, 52]. It is a phytoestrogen known to bind equally to estrogen receptors α and β [22, 51] and structurally similar to synthetic estrogens. The estrogenic agonist activity of resveratrol depends on the ERE sequence and the type of ER as well [19]. Transgenic studies revealed that the ERa subtype mediates sexual behavior, while ERb is more directly implicated in ovarian development [52].

Resveratrol can exert different actions in different cell types. The anti-proliferative activities of resveratrol may arise from its ability to interfere with nuclear factor-κB (NF-κB), p38 MAPK and phosphatidylinositol 3-kinase/Akt survival pathways [53], resulting in the suppression of DNA synthesis and cell proliferation, the inhibition of cell cycle progression and the induction of apoptosis. It is also important to note that the effects of RES on cellular growth are not universally inhibitory and, in several biological systems, RES has been shown to protect cells from death [54–59]. Therefore, the possibility of increasing the effectiveness of RES by associating vitD may be of clinical relevance in conditions linked to theca-interstitial cell hyperplasia, androgen excess and abnormal angiogenesis, such as PCOS, targeting most of the endocrine and metabolic underpinnings of PCOS. In PCOS, the typically enlarged ovaries are characterized by thecal and stromal hyperplasia [60]. This ovarian enlargement is associated with excessive ovarian androgen production and the disruption of menstrual cyclicity. Improvement of ovarian function, with restoration of ovulation and fertility, was observed with surgical reduction of ovarian size and/or partial destruction of ovarian tissues by procedures such as wedge resection and laparoscopic ovarian drilling [61, 62].

In addition to the new findings on essential molecular targets and signaling mechanisms triggered by RES and vitD, another important information is about bioavailability. The issue of bioavailability is determined by its rapid elimination and the fact that despite its highly effective absorption, the first hepatic step leaves little free RES. Indeed, only free RES can even bind to plasma proteins that could serve as a reservoir [8]. The in vivo phase of this study has shown that in ovarian tissue, RES exerts its effects in a cooperative manner with vitD. Specifically, in rat, RES in combination with vitD showed: a) a biphasic absorption rate not only in the ovary but also in the heart, kidney and liver tissues, related to blood concentration; b) increased bioavailability and biodistribution; c) reduced ROS production confirmed by SOD activity; d) modulation in a time-dependent manner of the levels of Cyclin D1 sustaining tissue homeostasis; e) a cooperative effect through the involvement of the ERβ receptor and VDR. The transport into bloodstream of RES was nonlinear during time, suggesting metabolism to be rate-limiting with respect to bioavailability. The second peak of plasma level after the oral dose may be due to enteric recirculation of conjugated metabolites by reabsorption after intestinal hydrolysis. In general, the doses of RES have been higher in animals than in humans. However, as in humans, the oral bioavailability in animals seems to be low and the metabolism involves both glucuronidation and sulfation [10]. For these reasons the dose of RES considered effective in human has been kept [24].

The execution of animal experiments is justified by the need of studying the rate of absorption of RES. After administration, RES undergoes a glucuronidation. There is evidence that the major form of RES transferred across the rat intestinal epithelium into the bloodstream is its glucuronide metabolite [63]. Therefore, an efficient carrier system should drive RES through the epithelial stratum to the bloodstream thus shortening its permeation time and metabolic turnover [7]. So, the aim of these additional experiments was to demonstrate that the effects observed in the in vivo experiments could be related with the previously observed in vitro effects with RES plus vitD.

Another novelty in this study is the observation that the cooperative mechanism has also been demonstrated in the intestinal absorption phase. This is clearly stated in a new set of in vivo experiments where the intracellular activated cascade mechanism after absorption demonstrates the cooperative mechanism. It is important to note that until now it was assumed that the RES also acted as a VDR agonist, but primarily in anti-tumor mechanisms [30].

Due to its estrogenic action, RES appears to be an optimal candidate for use in gynecological diseases, especially in the treatment of hot flashes (HF) associated with menopause. Vasomotor symptoms (VMS), including the hot flush, are amongst the commonest symptoms of the menopause transition period. Hot flushes are a heat dissipation response characterized by flushing and sweating, probably triggered by a narrowing of the thermoneutral zone in the hypothalamus and an increased central secretion of noradrenaline. The neuroendocrine changes associated with a hot flush may have significance far beyond the immediate distress and discomfort experienced at the time [64]. Despite various therapeutic solutions for the treatment of HF have been proposed, the results obtained do not show evidence of effectiveness in the use of phytoestrogens [65]. Although there are no human studies regarding the effects of resveratrol on menopausal signs and symptoms, a recent trial demonstrated that resveratrol may enhance mood and cognition in postmenopausal women [66]. Indeed, RES is effective in reducing the number of vasomotor episodes and the intensity of HF, with the transition from moderate/severe to mild symptoms in 78.6% of patients. Resveratrol has the characteristics to be an alternative therapy in the treatment of HF in menopause [24]. RES seems to act as an agonist/antagonist mixed α and β estrogen receptors. In fact, it binds β and α receptors with a comparable affinity, yet lower compared to estradiol. RES is different from other phytoestrogens, which bind the β receptor with greater affinity than the α receptor. Furthermore, it shows an estradiol antagonist behavior only to the α receptor. This would explain the beneficial effect of RES in the gynecological therapy [24].

Summing up, RES has been the focus of many recent in vitro and in vivo studies because of its pleiotropic biological activities [51, 67]. Its small molecular structure and polyphenolic character provides RES with antioxidant properties and multiple biological activities that are well documented when studied in vitro. However, some discrepancies have been observed in in vivo studies where effects may have low magnitude, mainly because of the limited distribution in tissues [8]. For this reason, research on RES uptake, cellular destination, metabolism and stability of the natural compound and of its metabolites as well is needed to elucidate its biological activity and it would be crucial to take advantage from its noteworthy properties [68]. For this reason, the scientific community is looking for innovative strategies to implement the bioavailability through drug delivery systems such as the use of nanoemulsion-based delivery systems [69] or through the ability to interact in a cooperative way with other molecules such as vitD.

However, the interplay between resveratrol and vitamin D must be further elucidated if the true potential of their clinical applications is to be revealed.

## Conclusions

In conclusion, this study demonstrated for the first time a cooperative effect of RES and vitD on ovarian cell and tissue, mediated by main physiological intracellular mechanisms. Such results could be used as a fundamental data for the development of new therapies for gynecological conditions, such as menopause-related hot-flashes**.**

## Additional file

Additional file 1: Additional details about RES quantification using HPLC-MS in cells and plasma samples. (DOCX 69 kb)

## Abbreviations

BBB: Blood-brain barrier
CHO-K1: Chinese Hamster Ovary cells
DMEM: Dulbecco’s modified Eagle’s medium
ER: Estrogen receptor
ERα: Estrogen receptor α
ERβ: Estrogen receptor β
FBS: Fetal bovine serum
FDA: Food and drug administration
HF: Hot flash
IL-6: Interleukin-6
MTT: 3-(4,5-dimethylthiazol-2-yl)-2,5-diphenyltetrazolium bromide
PCOS: Polycystic ovary syndrome
PMSF: Phenylmethanesulfonyl fluoride
PVDF: Polyvinylidene difluoride
RES: Resveratrol
ROS: Radical oxygen species
SOD: Superoxide dismutase
VDR: Vitamin D receptor
vitD: Vitamin D3
