# Supplementary material for: Biological effects of combined resveratrol and vitamin D3 on ovarian tissue
Source: J Ovarian Res. 2017 Sep 15;10:61. doi: 10.1186/s13048-017-0357-9 (PMC5602920; doi:10.1186/s13048-017-0357-9)
Supplement: Additional file 1: — Additional details about RES quantification using HPLC-MS in cells and plasma samples. (DOCX 69 kb) [file 13048_2017_357_MOESM1_ESM.docx]

**Additional file 1: Biological effects of Resveratrol and vitamin D3 combined on ovarian tissue**

**S1: HPLC-UV method for RES quantification in CHO-K1 cells.**

A Shimadzu HPLC system (Shimadzu, Kyoto, Japan), consisting of two LC-10AD Vp module pumps, an SLC-10A Vp system controller, an SIL-10AD Vp autosampler, and a DGU-14-A on-line degasser was used for the analysis. All the chromatographic separations were performed on a Phenomenex Luna PFP(2) 5µm (150 x 4.6 mm i.d.) (Torrance, CA, USA) as a stationary phase protected by a Luna PFP(2) SecurityGuard (Phenomenex). The SPD-M10Avp photodiode array detector was used to detect RES (306 nm). LC Solution 1.24 software was used to process the chromatograms. Aliquots (20 µL) of supernatants obtained from incubations were injected onto the HPLC system and eluted with a mobile phase (flow rate: 1 mL/min) consisting of solvent A 0.2% formic acid aqueous solution and solvent B 0.2% formic acid in acetonitrile. The following gradient elution was used: 0 min B=20%, 7 min B=80%, 9 min B=80%, 9.5 min B=20%, 15.5 min B=20%. The eluants were filtered through a 0.45-µm pore size nylon membrane filter before use.

**Figure S1.** Example of HPLC-UV chromatogram: RES, *Rt*= 7.7 min.

**S2: HPLC-MS method for RES quantification in rat plasma and tissues.**

A Thermo Finningan LCQ Deca XP plus system equipped with a quaternary pump, a Surveyor AS autosampler, a Surveyor photodiode array detector and a vacuum degasser was used for LC-MS analyses (Thermo Electron Corporation, Waltham, MA). Chromatographic separations was performed on a Kinetex Biphenyl (100 x 2.1 mm i.d.) (Torrance, CA, USA) as a stationary phase protected by a Kinetex Biphenyl SecurityGuard (Phenomenex). Aliquots (5µL) of samples were injected onto the HPLC system and eluted with a mobile phase (flow rate 250 µL/ min) consisting of solvents A (0.2% formic acid aqueous solution), and B (0.2% formic acid in acetonitrile). The following gradient elution was used: 0 min B=60%, 1 min B=65%; 2 min B=95%, 8 B=95%, 8.5 min B=60%, 13 min B=60%. The eluate was injected into the electrospray ion source (ESI) and MS spectra were acquired and processed using Xcalibur^®^ software. The operating conditions on the ion trap mass spectrometer were as follows: Positive mode: spray voltage, 5.30 kV; source current, 80 µA; capillary temperature, 300 °C; capillary voltage, 17.00 V; tube lens offset, 0.00 V; multipole 1 offset, -6.00 V; multipole 2 offset, -8.50 V; sheath gas flow (N_2_), 60 Auxiliary Units. Negative mode: spray voltage, 4.00 kV; source current, 80 µA; capillary temperature, 350 °C; capillary voltage, -9.00 V; tube lens offset, -5.00 V; multipole 1 offset, 6.25 V; multipole 2 offset, 19.50 V; sheath gas flow (N_2_), 60 Auxiliary Units. Data were acquired in positive full-scan and single reaction monitoring (SRM, 928🡪694) modes using mass scan range *m/z* 150-950. The collision energy was optimized at 33%.

**Figure S2.** Example of HPLC-MS chromatogram: RES-Dns, *Rt*= 6.9 min.

**S3: Chemical derivatization of RES with dansyl chloride (Dns-Cl).**
